# Outcomes of HIV-positive patients with non-tuberculous mycobacteria positive culture who received anti-tuberculous treatment in Botswana: Implications of using diagnostic algorithms without non-tuberculous mycobacteria

**DOI:** 10.1371/journal.pone.0234646

**Published:** 2020-06-12

**Authors:** Tefera Agizew, Rosanna Boyd, Unami Mathebula, Anikie Mathoma, Joyce Basotli, Christipher Serumola, Sherri Pals, Alyssa Finlay, Phenyo Lekone, Goabaone Rankgoane-Pono, Thato Tlhakanelo, Violet Chihota, Andrew F. Auld

**Affiliations:** 1 U.S. Centers for Disease Control and Prevention (CDC), Gaborone, Botswana; 2 Department of Family Medicine and Public Health, Faculty of Medicine, University of Botswana, Gaborone, Botswana; 3 School of Public Health, Faculty of Health Sciences, University of the Witwatersrand, Johannesburg, South Africa; 4 Division of Tuberculosis Elimination, CDC, Atlanta, Georgia, United States of America; 5 Division of Global HIV/AIDS and Tuberculosis, CDC, Atlanta, Georgia, United States of America; 6 Ministry of Health and Wellness, National Tuberculosis Control Programme, Gaborone, Botswana; 7 Aurum Institute, Johannesburg, South Africa; The University of Georgia, UNITED STATES

## Abstract

**Background:**

Patients with non-tuberculous mycobacteria (NTM) or *Mycobacterium tuberculosis* (MTB) pulmonary disease may have similar clinical presentation. The potential for misdiagnosis and inappropriate treatment exists in settings with limited testing capacity for Xpert^®^ MTB/RIF (Xpert), phenotypic culture and NTM speciation. We describe treatment outcomes among people living with HIV (PLHIV) who received anti-tuberculosis treatment and were found to have NTM or MTB positive sputum cultures.

**Methods:**

PLHIV attending one of the 22 participating HIV clinics, who screened positive for ≥1 tuberculosis (TB) symptoms (cough, fever, night sweats, or weight loss) were asked to submit sputa for culture and speciation from August 2012 to November 2014. The national intensified TB case finding algorithms were followed: initially symptomatic patients were evaluated by testing sputum samples using a smear (smear-based TB diagnostic algorithm) and, after GeneXpert instruments were installed, by testing with Xpert (Xpert-based TB diagnostic algorithm). Within the study period, TB diagnostic algorithms used for MTB did not include screening, diagnosis, and management of NTM. Despite MTB negative culture, some symptomatic patients, including those with NTM positive culture, received empirical anti-TB treatment at the discretion of treating clinicians. Per the World Health Organization treatment outcomes classification: died, treatment failure or loss-to-follow-up were classified as unfavorable (unsuccessful) outcome; cured and treatment completed were classified as favorable (successful) outcome. Empiric treatment was defined as initiating treatment without or before receiving a test result indicating MTB. We compare treatment outcomes and characteristics among patients with NTM or MTB positive culture who received anti-TB treatment.

**Results:**

Among 314 PLHIV, who were found co-infected with TB, 146 cases had microbiological evidence; and for 131/146 MTB positive cultures were reported. One-hundred fifty-two of the 314 were clinically diagnosed with TB and treated empirically. Among those empirically treated for TB, 36/152 had culture results positive for NTM, and another 43/152 had culture results positive for MTB, reported after patients received empirical anti-TB treatment. Overall, MTB positive culture results were reported for 174 (131 plus 43) patients. Treatment outcomes were available for 32/36 NTM and 139/174 MTB; unfavorable outcomes were 12.5% and 8.7% for NTM and MTB, respectively, *p = 0*.*514*, respectively. For 34/36 tested NTM patients, all Xpert results indicated ‘no MTB’. Among patients who initially received empiric anti-TB treatment and ultimately were found to have MTB positive culture, the unfavorable outcome was 11.8% (4/34), compared to 12.5% (4/32) of patients with NTM positive culture, Fisher’s exact test *p = 1*.*00*.

**Conclusions:**

While the higher unfavorable outcome was non statistically significant, the impact of inappropriate treatment among NTM patients should not be overlooked. Our findings suggest that Xpert has the potential to rapidly rule-out NTM and avoid sub-optimal treatment; further research is needed to evaluate such potential.

## Introduction

Patients with non-tuberculous mycobacteria (NTM) or *Mycobacterium tuberculosis* (MTB) pulmonary disease may have a similar clinical presentation with at least one of the World Health Organization (WHO) defined symptoms (cough, fever, night sweats, and weight loss), especially among people living with HIV (PLHIV) [1]. Amongst PLHIV, emerging evidence suggests that the rate of NTM diseases is increasing in Africa and other parts of the world [2, 3].

Where testing capacity for Xpert MTB/RIF, phenotypic culture and NTM speciation is limited, misdiagnosis of MTB or NTM may occur, resulting in inappropriate treatment and poor outcomes, particularly among patients with NTM disease. Recent report by Adikaram indicated that 4.5 to 15% of patients with NTM pulmonary disease have been erroneously diagnosed as having MTB [4]. Xpert MTB/RIF has high (>99%) specificity to rule-out MTB. The specificity among symptomatic patients with NTM positive culture, however, ranges from 92 to 99% [5–7] with a possibility of higher Xpert MTB/RIF false positivity [8].

After the WHO endorsement in 2010, the Botswana Ministry of Health and Wellness adopted the WHO guidelines and incorporated Xpert MTB/RIF into the national TB diagnostic algorithms in 2011. In Botswana, screening, diagnosis, and management of NTMs are not part of TB diagnostic algorithms [9].

The Xpert MTB/RIF Package Rollout Evaluation Study (XPRES) provided a unique opportunity to: (1) assess risk factors associated with increased likelihood of receiving anti-TB treatment among PLHIV with NTM; (2) describe treatment outcomes among PLHIV with NTM or MTB positive culture who received anti-TB treatment, and (3) compare treatment outcomes among PLHIV who initially received empirical anti-TB treatment and ultimately were found to have MTB positive culture versus NTM positive culture.

## Methods

### Study design and populations

This is a sub-study of the XPRES. Full details of the study protocol, including study populations, sample size, and procedures can be accessed in the published protocol. Except for prisoners, all PLHIV who consented and registered for HIV care for the first time [Antiretroviral therapy (ART) naïve] at the HIV care and treatment facility within the study period were eligible for enrollment [10].

In Botswana, as part of the national Xpert MTB/RIF roll-out, XPRES enrollment began in August 2012. XPRES included intensified active case finding activities and strengthening HIV patient retention interventions at 22 HIV treatment clinics before and after phased implementation of 13 GeneXpert instruments [10].

### Tuberculosis screening

At enrollment and each follow-up visit (i.e., at two weeks, then monthly for the first three months and then quarterly for the remaining follow-up period), adults and adolescents (combined into one adult group and defined as persons >12 years) and children (0–12 years old) were screened for TB symptoms. Per protocol, adults were screened for one or more of the four TB symptoms (cough, fever, night sweats, and weight loss) of any duration. Children were screened for weight loss or failure to thrive (no weight gain for > 3 months), cough for ≥ 2 weeks, fever for ≥ 2 weeks, fatigue/reduced playfulness for ≥ 2 weeks and profuse night sweats for ≥ 2 weeks [11]. Presumptive TB was defined when patients screened positive for one or more of the four TB symptoms.

### Sputum collection and diagnostic tests

Patients who were screened positive for one or more of the four TB symptoms were requested to provide four sputum samples, two were provided on the same day (Spot 1 and 2) and two on the following day. Day 2 sputa included one early morning sputum collected at home (Morning sample) and another sample at the clinic (Spot 3). Detailed laboratory procedures used for this study were described in a previous publication [1]. In summary, sputum samples were treated with BD Mycoprep (Beckton Dickinson, Sparks, Maryland, United States of America (USA)) which consists of 1% N-acetyl-L-cysteine (NALC), 4% sodium hydroxide and 2.9% sodium citrate, and then incubated in the automated BACTEC MGIT 960 instrument (Becton Dickinson, Sparks, Maryland, USA) [1].

Samples that failed to show growth after 42 days of incubation in the MGIT 960 were removed and classified as negative based on the manufacturer protocol. Samples exhibiting positive growth were removed from the instrument and inoculated on a blood agar to check for non-mycobacterial contamination. A Ziehl-Neelsen staining (ZN) smear was performed to check for the presence of Acid Fast Bacilli (AFB) [1].

Samples with positive growth in the MGIT 960 and AFB positive ZN staining, were tested with a rapid TB immunochromatographic assay (SD-Bioline Ag MPT64 RapidTM assay, Standard Diagnostics, Kyonggi-do, Korea) to discriminate between NTM and MTB [1]. Samples with positive growth in the MGIT 960 and AFB positive, but that were negative for MTB using the SD-Bioline assay, were sub-cultured on Lowenstein Jensen (LJ) media. Those that subsequently grew on LJ medium were considered to be presumptive NTMs and were characterized to species level with LPA (GenoType CM and AS assays, Hain Lifescience, Nehren, Germany) according to manufacturer recommendations [1].

To describe the specificity of Xpert MTB/RIF in our setting, all patients who had culture positive isolates identified as NTM were additionally tested retrospectively using Xpert MTB/RIF [1].

### TB diagnostic and treatment algorithms and definitions of treatment outcomes

The national intensified TB case finding algorithms were followed for diagnosis of TB patients (algorithms can be accessed in the published XPRES protocol) [10]: initially, symptomatic patients were evaluated by testing sputum samples using smear (smear-based TB diagnostic algorithm); after GeneXpert instruments were installed, testing was conducted by Xpert MTB/RIF (Xpert-based TB diagnostic algorithm). Though the follow-up culture testing was not part of the national TB diagnostic algorithms, this testing was conducted for all presumptive TB patients enrolled in the main study (XPRES). After treatment initiation, follow-up sputum-smear microscopy tests were conducted per the national tuberculosis program guidelines (i.e., at the end of months 2, 3, and 5 or 6) [10].

In the present study, patients who received standard anti-TB treatment [two months of Isoniazid (H), Rifampicin (R), Pyrazinamide (Z) and Ethambutol (E); and four months of HR] and died, failed treatment or loss-to-follow-up were classified as unfavorable (unsuccessful) outcome; outcome of cured or treatment completed was classified as favorable (successful) outcome, per the WHO treatment outcomes classifications [12]. Some patients received anti-TB treatment empirically, defined as initiating treatment without or before receiving a test result indicating MTB [13]. Despite MTB negative culture, some symptomatic patients with NTM positive culture, also received empirical anti-TB treatment at the discretion of treating clinicians.

### Data collection

Data were collected using standardized case report forms (CRF) between August 2012 and November 2014, and were double-entered into a Clindex database (Fortress Medical Systems, Minneapolis, MN, USA). Inconsistencies were identified through logic checks, and once identified were checked against the original CRFs. Where possible, inconsistencies and missing data were corrected through a review of patient charts.

### Statistical analysis

Data were analyzed using STATA (StataCorp. 2015. *Stata Statistical Software*: *Release 14*. College Station, TX: StataCorp LP) [14]. We used a Chi-square test to describe and compare demographic, clinical and laboratory characteristics of patients; and to compare drug-sensitive TB treatment outcomes among patients with MTB positive culture results versus patients with NTM positive culture results and receivinfg first-line anti-TB treatment. STATA survey commands were used to adjust standard errors for within-facility correlation using a robust variance estimator. *P* values of <0.05 were considered statistically significant.

### Ethical considerations

The study protocol was approved by the Institutional Review Board (IRB) at the Botswana Health Research and Development Committee, Ministry of Health and Wellness, Gaborone, Botswana (May 16, 2012); the US Centers for Disease Control and Prevention, Atlanta, Georgia, United States of America (USA) (July 19, 2012); the University of Pennsylvania, Philadelphia, PA, USA (June 24, 2012), and the University of Witwatersrand, Johannesburg, South Africa (July 12, 2017). Written informed consent was obtained from adults (≥18 years old). For participants aged 7–17 years, assent was obtained in addition to consent obtained from a parent or guardian.

## Results

**Fig 1:**
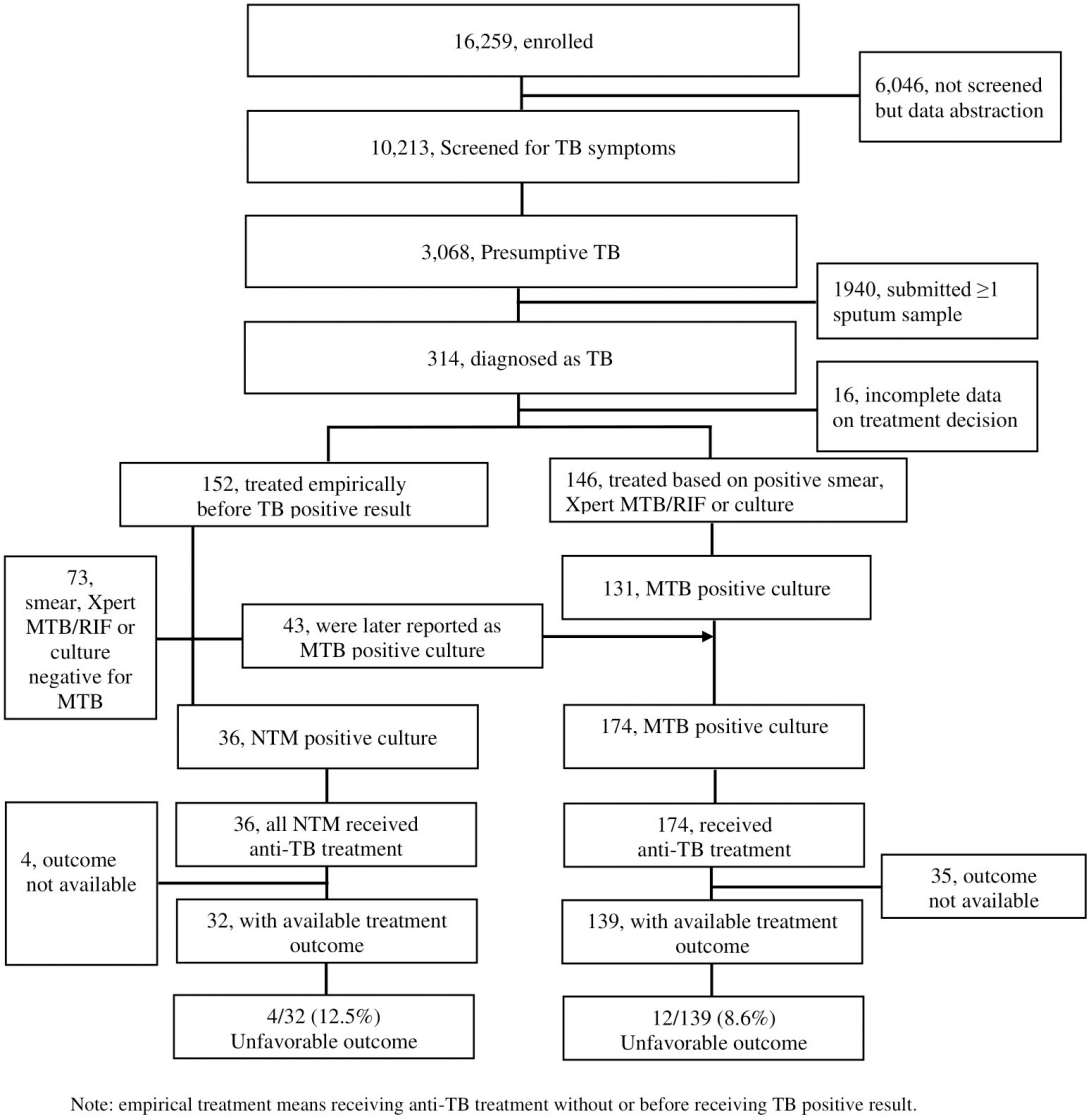
Treatment outcome among PLHIV with MTB or NTM positive culture in Botswana.

The enrolled patients were screened for one or more TB symptoms, submitted one or more sputum specimens, had available culture results (NTM and MTB) and received anti-TB treatment were reported in the previous XPRES publication [1]. In brief, 16,259 PLHIV were enrolled and 10,213 were screened for TB symptoms. For the remaining 6,046, data abstraction was conducted as outlined in an amendment to the main study’s (XPRES) data collection procedure. Among patients screened for TB symptoms, 30% (3,068/10,213) screened positive for ≥1 TB symptoms and 314 were diagnosed with TB. Of those diagnosed, 146 were based on microbiological evidence; for 131/146 patients, MTB positive culture results were reported. One-hundred fifty-two of 314 were clinically diagnosed as TB and were treated empirically. Sixteen of 314 had incomplete data on treatment decisions and were excluded from the analysis. For 36/152 patients, NTM positive culture results were reported. For another 43/152 patients, MTB positive culture results were reported after patients received empiric anti-TB treatment. Overall, MTB positive culture results were reported for 174 (131 plus 43) patients (See Fig 1).

Demographic (age < 35 years and gender), clinical (TB symptoms) and laboratory (CD4 cell count < 200 /mm^3^ and hemoglobin.) characteristics of patients receiving anti-TB treatment were similar among those with NTM positive culture results compared to those with MTB positive culture results. Among patients with NTM positive culture results, proportions with Body-Mass Index (BMI) <18.5 and previous history of TB were higher; however the proportion of those with night sweats was lower among NTM versus MTB positive culture results (Table 1).

**Table 1:** Characteristics of patients with NTM or MTB positive culture treated with an anti-TB treatment.

	NTM			MTB					
Characteristics	N	N	(%)	N	n	(%)	OR*	(95% CI)	*P* value**
**Age <35 years**	36	15	41.7	174	75	43.1	0.94	0.66–1.34	0.723
**Gender, female**	36	15	41.7	174	82	47.1	0.80	0.44–1.47	0.440
**CD4 cell count <200/mm**^3^	36	17	47.2	171	88	51.5	0.84	0.41–1.73	0.615
**BMI <18.5**	36	22	61.1	174	68	39.1	2.45	1.05–5.70	0.039
**Hgb <10 mg/dl**	29	5	17.2	151	46	30.5	0.48	0.17–1.37	0.152
**TB Symptoms**									
**Cough**	36	25	69.4	174	140	80.5	0.55	0.189–1.61	0.251
**Fever**	36	11	30.6	174	85	48.9	0.46	0.51–1.39	0.153
**Night sweats**	36	9	25.0	174	81	46.6	0.38	0.19–0.77	0.011
**Weight loss**	36	21	58.3	174	118	67.8	0.66	0.36–1.23	0.174
**History of TB, Yes**	35	10	28.6	174	23	13.2	2.63	1.04–6.66	0.043

NTM = Non-tuberculous mycobactera, MTB = Mycobacterium tuberculosis, OR = odds ratio, BMI = Body- Mass index and Hgb = Hemoglobin.

* OR was used with Chi-square when we adjusted for within-facility correlation using a STATA survey commad,

** *P* value adjusted for within-facility correlation.

### Species identification and Xpert MTB/RIF result for NTM isolates among patients treated with anti-TB treatment

NTM species for the 228 patients with NTM culture positive results were reported in a previous publication [1]. Table 2 displays the NTM species for the 36 patients with NTM who received anti-TB treatment. *M*. *intracellulare (42%*, *n = 15)* was the most common species isolated. Other NTMs commonly associated with pulmonary disease were *M*. *malmoense*, *M*. *simiae and* M. *asiaticum (each 6%*, *n = 2*); *M*. *avium*, *M*. *kansasii and M*. *lentiflavum (each 3%*, *n = 1)*. The common environmental contaminant *M*. *gordonae* was identified in 3 patients (8%). Eight (22%) NTMs could not be speciated by the current Hain GenoType CM and AS LPA that we used for testing. After excluding NTM isolates that were non-speciated, 89% (25/28) of the NTM isolates were potential pathogens (Table 2). Drug Susceptibility Testing (DST) was not conducted for all NTM positive cultures at the National TB Reference Laboratory. For all 36 patients in this analysis, isolates from the NTM positive culture specimens were tested retrospectively using Xpert MTB/RIF; all results showed ‘no MTB’, indicating one-hundred percent specificity. During the study period, the routine TB diagnostic algorithms in Botswana had no provision for further NTM screening, diagnosis or treatment, thus NTM specific treatment was not offered to any of the patients with NTM.

**Table 2:** NTM isolated among PLHIV presenting with TB symptoms who received anti-TB treatment in Botswana and corresponding Xpert MTB/RIF result.

		Xpert MTB/RIF*		
NTM Species	Number	Frequency	MTB detected	MTB not detected
*M*. *intracellulare*	15	42%	0	15
*M*. *gordonae*	3	8%	0	3
*M*. *malmoense*	2	6%	0	2
*M*. *simiae*	2	6%	0	2
*M*. *asiaticum*	2	6%	0	2
*M*. *avium*	1	3%	0	1
*M*. *kansasii*	1	3%	0	1
*M*. *lentiflavum*	1	3%	0	1
Mixed NTM**	1	3%	0	1
*Others*†	8	22%	0	6
Total	36	100%	0	34

* Xpert MTB/RIF test result was from spot 1 & 3.

** Mixed species: more than one NTM species identified per isolate.

^†^NTMs ‘that we were not able to speciate further using the current testing methods we used.

### Treatment outcomes among PLHIV with NTM or MTB positive culture treated with anti-TB treatment

Among 314 patients diagnosed with TB, culture results were positive for 210 (36 NTM and 174 MTB). All 36 patients with NTM and 174 of patients with MTB received first-line anti-TB treatment (two months of HRZE and four months of HR). TB treatment outcomes were available for 89% (32/36) of patients with NTM and 80% (139/174) of patients with MTB. The proportions of unfavorable outcomes were 12.5% (4/32) among patients with NTM and 8.6% (12/139), among patients with MTB, *p = 0*.*484*, Fisher’s exact test (Fig 1 and Table 3). Further analysis indicated no statistically significant difference in each of the unfavorable outcomes between patients with NTM and MTB. Compared to MTB, the proportion of patients who died in the NTM group was 9% (3/32) versus 4% (6/139), *P = 0*.*22*, Fisher’s exact test. There was no failure in either group, and loss-to-follow-up was 3% (1/32) versus 4% (6/139) between patients with NTM and MTB, respectively. It is worth noting that before the culture results were available sputum-smear microscopy and Xpert MTB/RIF results were used by treating clinicians. For 7/36 patients AFB was detected by smear, and for the 29 patients with available Xpert MTB/RIF results, MTB was not detected.

**Table 3:** Treatment outcomes among PLHIV with NTM or MTB positive culture treated with anti-TB treatment.

Anti-TB treatment outcomes	NTM n = 36	MTB n = 174	*P* value*
Unfavorable outcome	4 (12.5%)	12 (8.7%)	0.484
Favorable outcome	28 (87.5%)	127 (91.3%)	
Sub Total	32 (100%)	139 (100%)	
Transferred out or not evaluated	4	35	

The total patients with MTB positive culture were 180, 6 were loss-to-follow-up before TB treatment initiation.

* *P* value adjusted for within-facility correlation.

Treatment outcomes were not available for 11.1% (4/36) and 20.1% (35/174) of patients with NTM and MTB positive culture results, respectively. Considering the potential change of the overall result, we did sensitivity analysis using four scenarios for the missing outcomes: (1) If all 4 (100%) NTM and all 35 (100%) MTB patients with missing outcomes had unfavorable outcomes, the total unfavorable outcome proportion would be 22.2% (8/36) versus 27% (47/174), respectively, Odds ratio (OR), 0.77, *p = 0*.*552*, (2) If none of the 4 NTM and the 35 MTB patients with missing outcomes had unfavorable outcomes, the total unfavorable outcome would be 11.1% (4/36) versus 6.9% (12/174), respectively, Fisher’s exact test, *p = 0*.*486*, (3) If 2 (50%) of 4 NTM and 18 (50%) of 35 MTB patients with missing outcomes had unfavorable outcomes, the total unfavorable outcome would be 16.7% (6/36) versus 17.2% (30/174), respectively, OR, 0.96, *p = 0*.*934* and (4) Adjusting unfavorable outcomes for missing outcomes (4 for NTM and 35 for MTB) based on the actual proportion of unfavorable outcomes for NTM (12.5%) and MTB (8.6%). Using this method, the total unfavorable outcome would be 13.9% (5) for NTM [4 plus 1 (12.5% of 4 missed)] and 8.6% (15) for MTB [12 plus 3 (8.6% of 35)], OR, 1.71, *p = 0*.*327*. In all scenarios, no significant differences were noted, indicating that the effect of the missing outcomes on changing overall results would be unlikely.

Among 152 patients who received empiric anti-TB treatment, 43 were later confirmed to have MTB with positive culture results (See Fig 1). Treatment outcomes were available for 34/43 patients. The unfavorable outcome among the 34 patients was 11.8% (4/34), compared to 12.5% (4/32) of patients with NTM positive culture results and available treatment outcomes, Fisher’s exact test *p = 1*.*00*. Outcome of ‘died’ was 9% for both MTB (4/34) and NTM (3/32).

### Risk factors associated with increased likelihood of receiving anti-TB treatment among PLHIV with NTM

Among 228 NTM patients, those with BMI <18.5, weight loss and TB history had higher odds of receiving anti-TB treatment (Table 4).

**Table 4:** Risk factors associated with increased likelihood of receiving anti-TB treatment among PLHIV with NTM.

	NTM treated with anti-TB			NTM not treated with anti-TB					
Characteristics	N	n	(%)	N	n	(%)	OR*	(95% CI)	P value**
**Age < 35 years**	36	15	41.7	192	69	35.9	1.27	0.76–2.12	0.324
**Gender, female**	36	15	41.7	192	108	56.3	0.56	0.29–1.05	0.068
**CD4 cell count <200/ mm**^3^	36	17	47.2	184	80	43.5	1.16	0.69–1.95	0.537
**BMI <18.5**	36	22	61.1	178	48	27.0	4.26	2.49–7.29	<0.001
**Hgb <10 mg/dl**	29	5	17.2	162	25	15.4	1.14	0.45–2.87	0.759
**TB Symptoms**									
**Cough**	36	25	69.4	184	127	69.0	1.02	0.41–2.54	0.963
**Fever**	36	11	30.6	183	41	22.4	1.52	0.52–4.48	0.411
**Night sweats**	36	9	25.0	184	48	26.1	0.94	0.41–2.20	0.885
**Weight loss**	36	21	58.3	185	72	38.9	2.20	1.21–4.00	0.014
**History of TB, Yes**	35	10	28.6	183	17	9.3	3.91	1.17–13.0	0.030

NTM = Non-tuberculous mycobacteria, OR = odds ratio, BMI = Body-Mass Index and Hgb = Hemoglobin,

* OR was used with Chi-square when we adjusted for within-facility correlation using a STATA survey commad,

** P value adjusted for within-facility correlation.

## Discussion

The present analysis, to our knowledge, is the first study reporting on the potential likelihood of unfavorable treatment outcomes among PLHIV with NTM positive culture results who received standard anti-TB treatment, compared to patients with confirmed drug-sensitive TB. This study provides insight into the implications of not including NTM diagnostic and management standards into routine clinical practice. Thirty-six symptomatic patients with documented NTM positive culture reports received anti-TB treatment that might have been avoided. In these symptomatic patients with NTM positive culture results, there was no other laboratory evidence of MTB; all NTM isolates were additionally tested retrospectively using Xpert MTB/RIF; results indicated ‘no MTB’ (100% specificity). A non-significant, higher unfavorable treatment outcome (12.5% versus 8.6%) was observed among patients with NTM compared to MTB, respectively. This is a potential concern, though this report was developed from a sub-analysis that was not designed with sufficient power to address this objective.

In previous publications, data are limited on this topic and we were unable to directly compare to our study. All patients in this current anlaysis were HIV-positive. Maiga *et al* reported data from Mali, whereby 12% (17/142) of NTM positive culture results were amongthe both HIV-positive and HIV-negative patients. Six of the 17 were co-infected with MTB and HIV, and received standard anti-TB treatment. The remaining 11 were also treated with either second-line TB treatment or re-treatment regimen since they were considered as having chronic TB, despite MTB negative culture results. Furthermore, due to limited follow-up, treatment outcome was not ascertained for these 17 patients [15]. Thus the results from Mali were not directly comparable to our study.

In our analysis, we identified that symptomatic patients with NTM positive culture results, who were also negative for MTB by Xpert MTB/RIF received standard anti-TB treatment at the discretion of treating clinicians. This is alarming given the laboratory evidence was not in favor of using standard anti-TB treatment for such patients. Within the present study period, TB diagnostic algorithms in Botswana did not address patients with NTM [9]. As a result, none of the patients with NTM positive culture results received NTM specific treatment. The fact that the current TB diagnostic algorithms in Botswana do not include screening, diagnosis and management of patients with NTM disease indicates that clinicians might have not been equipped to resolve a dilemma of how to appropriately manage HIV-positive patients with TB symptoms and NTM positive culture results with no other alternative diagnosis [9]. It is worth noting that in relation to supporting clinical decisions, Xpert MTB/RIF negative results may be used in two ways: (1) Since the sensitivity of Xpert MTB/RIF for MTB in HIV-positive persons is not one-hundred percent, with strong clinical suspicious for TB, it may be reasonable to empirically treat an Xpert MTB/RIF negative symptomatic patients while awaiting culture results. If the culture is positive for MTB, treatment would continue; (2) If the culture is positive for NTM, consider cessation of empirical anti-TB treatment and investigate further for NTM species identification and treatment for NTM as appropriate [16]. Among PLHIV, the present study and other emerging evidence suggest that the rate of NTM is increasing in Africa and other parts of the world [2, 3]. The findings from these studies have important program implications, especially for HIV-positive patients with advanced stage of HIV disease. Because of the difficulty of distinguishing between NTM colonization and NTM disease, the American Thoracic Society (ATS) and Infectious Diseases Society of America criteria are recommended to diagnose NTM disease [17].

In previous XPRES publication, we acknowledged the potential clinical relevance of NTM colonization, infection, and disease. Though we have collected more than one sputa, our study was not designed to address NTM diagnosis per the ATS criteria; consequently, distinguishing NTM colonization versus disease was not possible [1]. Furthermore, the management of NTM disease is a challenge since it usually needs experienced clinicians equipped with reliable laboratory results for culture and DST. Additionally, depending on the NTM species, a combination of drugs are used for a prolonged period of time [18]. It is very clear that NTM infection and disease are a possibility in Botswana. The potential for unfavorable outcome is demonstrated in the present study when symptomatic patients with NTM positive culture are treated with anti-TB treatment. Thus the national TB programs, in Botswana and similar settings, should consider a modification of the TB diagnostic algorithms to cater for NTM infection and disease, especially among patients with TB symptom and negative Xpert MTB/RIF testing. Additionally, a curriculum for clinicians training on NTM screening, diagnosis, and appropriate NTM species-specific management is needed.

Though the tested samples (45 or less) were limited, the earlier studies showed specificity of Xpert MTB/RIF, ranging between 92–99% [5–7]. In 2017, Agizew *et al* reported the highest tested number of isolates (n = 219) that indicated a higher specificity (99%) to rule-out TB among NTM positive culture isolates [1]. Where testing capacity for Xpert MTB/RIF, phenotypic culture and NTM speciation is limited, misdiagnosis, delivery of sub-optimal treatment and the poor outcome may continue to occur, especially since up to 6% of patients with NTM positive culture may have AFB positive result with sputum-smear microscopy. The concern of managing AFB positive NTM patients with anti-TB treatment might be minimized if countries are shifting a smear-based diagnostic algorithm to Xpert MTB-based diagnostic algorithm [1].

Among patients with NTM positive culture results, BMI <18.5, weight loss and previous history of TB were identified as risk factors to be misdiagnosed as TB and receiving anti-TB treatment. In our analysis, out of the 36 patients with NTM positive culture results who received anti-TB treatment, 34 were additionally tested using Xpert MTB/RIF, and all test results indicated ‘no MTB’. Thus, when MTB is not detected with Xpert MTB/RIF among patients with NTM positive culture results, anti-TB treatment can be avoided. During clinicians’ training on TB diagnostic algorithms, trainers may want to emphasize how reliance on Xpert MTB/RIF result might reduce the potential risk of misdiagnosis of patients with NTM as TB, especially among patients with no strong clinical suspicion of TB. Furthermore, National TB programs, need to strengthen diagnostic capacity (such as Line probe assay, a molecular to identify NTM species) [19], include NTM screening, diagnosis and treatment in TB diagnostic algorithms. For presumptive TB patients with negative Xpert MTB/RIF result, additional culture testing needs to be performed. With a culture indicating NTM-positive, with further NTM species identification NTM species-specific treatment be initiated.

Our study has some limitations. First, since the study was not designed with enough power to address treatment outcomes among patients with NTM versus MTB our finding (a non-significant higher unfavorable outcome among NTM) may not be considered conclusive in a bigger picture. Second, the results analyzed were from symptomatic patients who were able to submit at least one sputum. Those patients who screened positive for one or more of the four TB symptoms but were not able to produce sputum were excluded and the proportion of potential pulmonary NTM infection may be underestimated as we indicated in our previous publication [1]. Third, low BMI, weight loss and previous history of TB were identified as risk factors for misdiagnosis as TB and receiving anti-TB treatment among patients with NTM positive culture results; and our data was not necessarily complete since we have not used the American Thoracic Society (ATS) and Infectious Diseases Society of America (IDSA) criteria [17]. Fourth, treatment outcomes were not available for 4 and 35 patients with NTM and MTB positive culture results, respectively. With these missing outcomes, there is a potential in changes of the overall result and conclusion. However, we did sensitivity analysis with the four scenariors as indicated in our results. While we acknowledge that these four scenarios might not be exhaustive, the sensitivity analysis indicates that significant changes in our overall results and conclusions would be less likely, if there were no patients with missing outcomes. Fifth, we have reported in our previous publication how we were able to assess and rule-out the potential contamination from water sources at our TB referral laboratory [1]; however, we were not able to rule-out the NTM colonization or environmental contamination from peripheral clinics where sputa were collected.

In conclusion, while the higher unfavorable outcome was non-significant, the effect of inappropriate treatment among NTM patients cannot be ruled out. Our findings highlight factors associated with increased likelihood of NTM patients receiving anti-TB treatment. NTM patients with BMI <18.5, weight loss and previous history of TB were more likely to receive anti-TB treatment inappropriately. Xpert has the potential to rapidly rule-out NTM and avoid sub-optimal treatment; further research is needed to evaluate such potential.
